# Identification of Suitable Reference Genes for Real Time Quantitative Polymerase Chain Reaction Assays on *Pectoralis major* Muscle in Chicken (*Gallus gallus *)

**DOI:** 10.1371/journal.pone.0127935

**Published:** 2015-05-28

**Authors:** Carlos S. Nascimento, Leandro T. Barbosa, Claudson Brito, Roberta P. M. Fernandes, Renata S. Mann, Ana Paula G. Pinto, Haniel C. Oliveira, Mike V. Dodson, Simone E. F. Guimarães, Marcio S. Duarte

**Affiliations:** 1 Department of Animal Science, Universidade Federal de Sergipe, São Cristovão, Sergipe, Brazil; 2 Department of Physiology, Universidade Federal de Sergipe, São Cristovão, Sergipe, Brazil; 3 Department of Crop Science, Universidade Federal de Sergipe, São Cristovão, Sergipe, Brazil; 4 Department of Animal Science, Universidade Estadual de Santa Cruz, Ilheus, Bahia, Brazil; 5 Department of Animal Science, Washington State University, Pullman, Washington, United States of America; 6 Animal Biotechnology Laboratory—LABTEC, Department of Animal Science, Universidade Federal de Viçosa, Viçosa, Minas Gerais, Brazil; University of Lleida, SPAIN

## Abstract

Thirteen reference genes were investigated to determine their stability to be used as a housekeeping in gene expression studies in skeletal muscle of chickens. Five different algorithms were used for ranking of reference genes and results suggested that individual rankings of the genes differed among them. The stability of the expression of reference genes were validated using samples obtained from the *Pectoralis major* muscle in chicken. Samples were obtained from chickens in different development periods post hatch and under different nutritional diets. For gene expression calculation the ΔΔCt approach was applied to compare relative expression of pairs of genes within each of 52 samples when normalized to *mitochondrially encoded cytochrome c oxidase II* (MT-CO2) target gene. Our findings showed that *hydroxymethylbilane synthase* (HMBS) and *hypoxanthine phosphoribosyl transferase 1* (HPRT1) are the most stable reference genes while *transferrin receptor* (TFRC) and *beta-2-microglobulin* (B2M) ranked as the least stable genes in the *Pectoralis major* muscle of chickens. Moreover, our results revealed that HMBS and HPRT1 gene expression did not change due to dietary variations and thus it is recommended for accurate normalization of RT-qPCR data in chicken *Pectoralis major* muscle.

## Introduction

Quantitative real-time PCR (RT-qPCR) is a gold-standard biotechnique for gene expression analyses but requires a robust reference genes to correct for technical variation within the experiment [1]. Expression data from RT-qPCR analysis requires reliable normalization to avoid false positive results that may lead to misinterpretations and imprecise conclusions. One of the major difficulties in obtaining accurate expression patterns is the removal of experimentally induced non-biological variation from the true biological variation. A widely used method for normalization involves measuring the expression of a reference gene [2]. A gene can be used as a reference when it is highly and stably expressed in all samples under investigation and is not co-regulated with a target gene [3]. Thus, normalization of gene expression based on a varying reference gene is likely to produce misleading results [4]. According to the Minimum Information for Publication of Quantitative Real-Time PCR Experiments (MIQE) guidelines, reference genes are now selected based on their specificity in interactions between a species or tissue subjected to different experimental treatments. Several studies have reported evaluations of reference gene in animals such as cattle [5], pig [6], sheep [7], goat [8], horse [9], fish [10], and birds [11]. However, a limited number of studies have investigated identification of reference genes used in chicken (*Gallus gallus*). Moreover, the systematic evaluations of reference genes for chicken specie other than the immune [12] and fibroblast [13] cells are scarce.

The expression stability of reference genes was investigated using five different algorithms. This has enabled us to assess the suitability of 13 common reference genes in *Pectoralis major* muscle. Samples were obtained from chickens in different development periods post hatch and under different nutritional diets Our results revealed that HMBS and HPRT1 gene are most stable and thus recommended for accurate normalization of RT-qPCR data while TFRC and B2M varied across the experimental conditions and thus are not recommended to normalize gene expression data.

## Material and Methods

### Ethical approval

All experiments were approved by the Universidade Federal de Sergipe Animal Ethics Committee and Brazilian national standards for animal research.

### Broiler, experimental condition and muscle sampling

A total of one hundred sixty-eight 1-day-old Cobb male broilers were obtained from a commercial hatchery after being vaccinated against Marek’s disease and Newcastle disease. Broilers were allocated in floor pens until day 7. The floor pen had wood shavings and was equipped with tube feeder and nipple drinkers. Temperature was maintained at 32°C at placement, and was gradually reduced to ensure comfort by using a thermostatically controlled heater and fans. To evaluate the stability of expression of the reference genes tested, broilers were submitted to different diets, and slaughtered at different days. At 8 d post hatch, animals were randomly assigned into four groups with seven replicate cages of six birds each. Broilers were fed ad libitum four different growing diets and subsequently four finishing diets for 14 d each. Experimental diets were based on corn and soybean meal and were formulated using the food chemical composition values and nutritional requirements for the average performance of male broilers [14]. Experimental diets were maize-soyabean meal-based supplemented with four levels of L-Lys (12.71, 11.82, 10.99 or 10.22 g/kg) during the growing phase, and four levels of L-Lys (11.25, 10.46, 9.72 or 9.030 g/kg g/kg) during the finishing phase. A total of 52 broilers (4 male in each group) were randomly sampled at 8, 21, 35 and 42 days post hatch and slaughtered. The control group was composed by broilers supplemented with 0 g/kg of L-Lys and slaughtered at 8 d post hatch. Samples of *Pectoralis major* muscle were isolated and placed in sterile tubes containing RNAlater (Ambion, RNA Carlsbad, CA, USA). Samples were placed at 4°C for 12 h and then stored at 70°C prior to total RNA isolation.

### RNA isolation and cDNA synthesis

Total RNA was isolated using the SV Total RNA Isolation System Kit (Promega, Madison, WI, USA) according to the manufacturer’s protocol. The RNA concentration was determined by analyzing 2.5 μl of extract using AstraGene UV/Vis Spectrophotometer (AstraNet Inc., Bath, UK). Integrity of RNA was evaluated using 1% agarose gel. As quality control assay, the absence of contaminant genomic DNA in RNA preparations was verified using RNA as a template in real-time PCR assays (minus RT control, i.e. RNA not reverse-transcribed to cDNA). One microgram of total RNA from each group was subjected to oligo (dT) reverse transcription using the GoScript Reverse Transcription System (Promega, Madison, WI, USA) following the manufacturer’s instruction. The resulting cDNA samples were stored at -20°C until RT-qPCR analysis was performed.

### Selection of reference genes and RT-qPCR optimization

**Fig 1:**
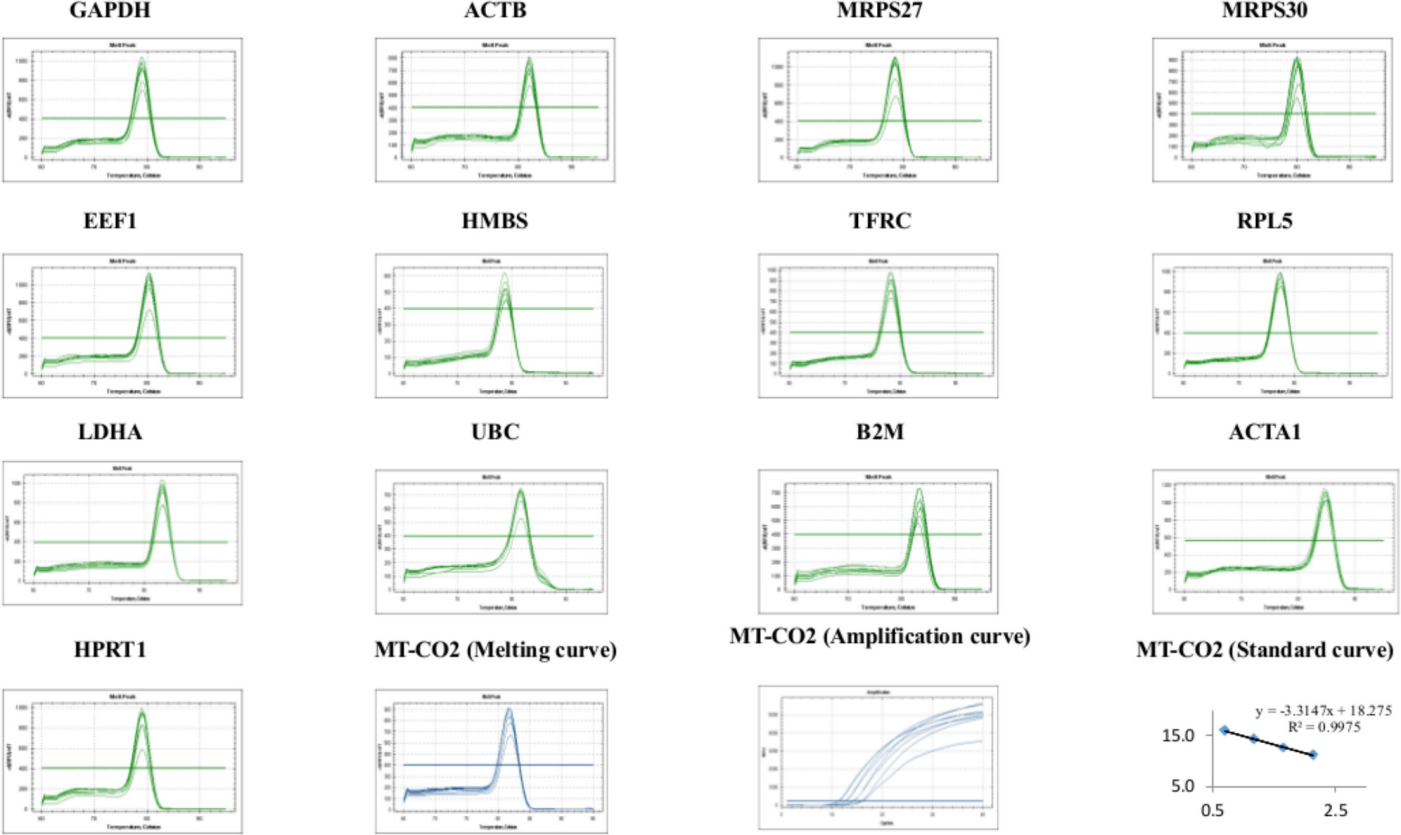
Specificity of RT-qPCR amplification. Melting curves (dissociation curves) of the 13 reference genes amplicons after the RT-qPCR reactions, all showing one peak. Melting, amplification and standard curves for *mitochondrially encoded cytochrome c oxidase II* (MT-CO2) target gene are also showed.

Thirteen reference genes were selected for investigation to identify the most stably expressed reference gene(s) to be used in RT-qPCR studies. Reference genes were chosen based on literature and nucleotide sequences were recovered from the Ensembl database (www.ensembl.org/). These sequences were used to design primers by the PrimerQuest software provided by Integrated DNA Technologies, Inc (IDT, Coralville, IA, USA). Additionally, BLAST analyses were performed to verify their specificity. The primers sequence information used on this study is shown in Table 1 and the primer specificity in the Fig 1.

**Table 1:** Reference genes, MT-CO2 target gene, specific RT-qPCR primers and different parameters derived from RT-qPCR analysis.

Gene symbol	Transcript ID	Description	Primer sequences (5′-3′)	Ta(°C)	Amplicon size (bp)
ACTA1	ENSGALE00000120039	*Actin*, *Alpha 1*, *Skeletal Muscle*	F: CTCCGGCGATGGTGTGA	62	122
			R: CAGTCAGGATCTTCATCAGGTAGT		
ACTB	ENSGALT00000015673	*Actin*, *beta*	F: ACCCCAAAGCCAACAGA	60	136
			R: CCAGAGTCCATCACAATACC		
B2M	Z48922	*beta-2-microglobulin*	F: CCACCCAAGATCTCCATCAC	62	90
			R: CGTCCAGTCGTCGTTGAA		
EEF1	NM_204157.2	*Eukaryotic translation elongation factor 1 alpha 2*	F: GCCCGAAGTTCCTGAAATCT	60	102
			R: AACGACCCAGAGGAGGATAA		
GAPDH	ENSGALE00000024703	*Glyceraldehyde-3-phosphate dehydrogenase*	F: CTGTAGCCCATATCTTGCCTTT	60	95
			R: CAAGACGATCTCCACTCTTTCC		
HMBS	ENSGALE00000001922	*Hydroxymethylbilanesynthase*	F: TGACCTGGTAGTTCACTCCTT	60	75
			R: TTGCAAATAGCACCAATGGTAAAG		
HPRT1	AJ132697	*HypoxanthinePhosphoribosyltransferase 1*	F: GCACTATGACTCTACCGACTATTG	60	112
			R: CAGTTCTGGGTTGATGAGGTT		
LDHA	ENSGALE00000067556	*Lactatedehydrogenase A*	F: CTATGTGGCCTGGAAGATCAG	60	124
			R: GCAGCTCAGAGGATGGATG		
MRPS27	XM_424803	*Mitochondrialribosomalprotein S27*	F: GCTCCCAGCTCTATGGTTATG	60	124
			R: ATCACCTGCAAGGCTCTATTT		
MRPS30	NM_204939.1	*Mitochondrialribosomalprotein S30*	F: CCTGAATCCCGAGGTTAACTATT	60	107
			R: GAGGTGCGGCTTATCATCTATC		
RPL5	NM_204581.4	*Ribosomalprotein L5*	F: AATATAACGCCTGATGGGATGG	60	99
			R: CTTGACTTCTCTCTTGGGTTTCT		
TFRC	ENSGALE00000080099	*Transferrin receptor (p90*, *CD71)*	F: CTCCTTTGAGGCTGGTGAG	60	89
			R: CGTTCCACACTTTATCCAAGAAG		
UBC	M11100.1	*Ubiquitin C*	F: CACCCTGTCTGACTACAACATC	62	92
			R: ACAAGACTGCTGACAACAACTA		
MT-CO2	ENSGALT00000029090	*Cytochrome C Oxidase II*	F:GTCCTCATTACTGCCATCCTAC	62	115

Prior to the RT-qPCR amplification, part of cDNA was pooled into a new tube to make one composed sample which was then diluted to construct the standard curves for PCR conditions optimization and calculation of PCR efficiency. For this purpose, four cDNA quantity (1, 5, 15, 45 ng) and four primers concentrations (200, 400, 800 and 1000 nM) were tested. The following experimental RT-qPCR conditions were used: 1 cycle of 95°C for 10 min, 40 cycles of 95°C for 10 sec and 60 sec at 60–62°C. Additional steps with a gradual increase in temperature from 60–62 to 95°C were used to obtain the dissociation curve. After the analysis of efficiency, the most adequate annealing temperature and primer concentration was used to perform PCR reaction. A reaction mix without template was also used to detected possible reagent contamination. The PCR amplification reaction was performed at different wells and in duplicates. Each primer pair was checked for size specificity of the amplicon by 1.5% agarose gel electrophoresis and ethidium bromide staining. The PCR amplification efficiencies were calculated for each target and reference gene assay using the formula E = (10-1/slope—1) × 100 [15].

### Quantitative real-time PCR and preprocessing

Quantitative real-time PCR reactions were performed using SYBR Green detection with GoTaq qPCR Master Mix (Promega, Madison, WI, USA), using gene specific primers. All reactions were performed on CFX96 Real-Time PCR Detection System. A blank (No Template Control) was also added in each assay. The thermal cycling conditions for the RT-qPCR were as follows: 1 cycle at 95°C for 10 min, 40 cycles of amplification at 95°C for 15 s and annealing at 60–62°C for 1 min. The average of the quantification cycle (Cq), defined as the fractional cycle number at which the fluorescence passes the fixed threshold, was determined using manual quantification settings. Values of Cq for control wells were excluded from further analysis, as the values were greater than 35 or not detected.

### Determination of expression stability of reference genes

To prepare data set input, Cq values were transformed to relative quantities using the formula (1+E)^-∆Cq^ [16]. The sample with Cq maximum expression was used as the calibrator with a set value of 1 ((1+E)^-Cqoriginal—Cqmax^). Thus, the lowest value was zero. To calculate the stability of the candidate genes we have used the BestKeeper [17], NormFinder [18], geNorm Excel [19], geNorm SAS [20] and ΔCt method [21] were used. Relative quantities was used as the input to perform the analysis on NormFinder, geNorm and ΔCt method. No transformed Cq values are required for BestKeeper analysis. In addition, geNorm was used to determine the optimal number and the reference genes required for reliable normalization of RT-qPCR data. All data were analyzed according to the instructions for each software tested.

### Relative expression of MT-CO2 gene

Relative expression of MT-CO2 target gene was normalized by the reference genes through Pfaffl method. The relative expression of target gene was calculated from the geometric average of two best and worst reference genes. The geometric average of the Cq values with a standard deviation of less than 0.5 cycles was used in further calculations. Significance values were obtained by using analysis of variance (ANOVA) for all the tested reference genes between different contrasting groups. Relative expression at 8 days was used as control. A “t” test was applied to detect statistical difference between mean of groups and differences were considered at α = 0.05.

## Results

### Primer efficiency and specificity

The annealing temperature of 60–62°C was effective for all primers. The RT-qPCR assays had amplification efficiencies between 91% and 104%. The determination coefficients (R^2^) values were higher than 0.9909 except for UBC (0.9705; Table 1). Primer specificity was evaluated with a single peak in all melting curves and no primer-dimer was detectable indicating optimal performance of the primers (Fig 1). The no-template controls (NTC) that was included in all RT-qPCR assays did not show any positive Cq value throughout the study.

### Comparing reference genes by descriptive statistics

A RT-qPCR assay based on SYBR Green detection was designed for the transcriptional profiling of 13 genes commonly used as reference in experiments of RT-qPCR analysis (Table 2). Evaluation of quantification cycle (Cq) values by descriptive statistical analysis showed a variability among the different reference genes. The 13 genes were clearly distributed into two different categories of expression level. Six genes (ACTA1, EEF1, GAPDH, LDHA, RPL5, UBC) coded for highly expressed mRNAs with the majority of Cq values between 13 and 20 cycles; seven other genes (ACTB, HMBS, HPRT1, MRPS27, MRPS30, TFRC, B2M) were moderately expressed with Cq values between 21 and 28 cycles (Table 2). As shown in the Table 2, MRPS27 (CV = 2.27%) and HPRT1 (CV = 2.34%) had the narrowest variance (lowest Cq dispersion), while TFRC (CV = 5.18%) and B2M (CV = 4.57%) had the widest variance (highest dispersion).

**Table 2:** Descriptive statistics and expression level of reference genes obtained by BestKeeper (n = 52).

Factor	ACTA1	ACTB	B2M	EEF1	GAPDH	HMBS	HPRT1	LDHA	MRPS27	MRPS30	RPL5	TFRC	UBC
geoMean [Cq]	13.88	21.51	23.40	19.83	17.21	26.46	24.27	15.92	24.70	25.91	19.53	28.49	18.13
arMean [Cq]	13.89	21.53	23.44	19.85	17.23	26.48	24.28	15.93	24.71	25.93	19.54	28.55	18.15
min [Cq]	12.37	18.30	20.47	17.63	14.67	24.96	22.61	14.90	23.30	24.31	18.26	24.24	16.61
max [Cq]	16.28	24.32	27.13	21.69	19.64	29.31	26.42	18.23	26.61	28.75	21.29	32.81	21.85
**SD [± Cq]**	**0.531**	**0.812**	**0.883**	**0.634**	**0.6724**	**0.640**	**0.568**	**0.624**	**0.561**	**0.637**	**0.484**	**0.435**	**1.401**
**CV [% Cq]**	**3.826**	**3.773**	**4.992**	**3.194**	**3.901**	**2.418**	**2.339**	**3.916**	**2.269**	**2.456**	**2.479**	**2.244**	**4.970**
Coeff. of corr. [r]	0.92	0.88	…	0.91	0.91	0.96	0.86	0.88	0.91	0.94	0.91	. . .	. . .

Abbreviations: Cq: quantification cycle; GM [Cq]: geometric Cq mean; AM [Cq]:arithmetic Cq mean; Min [Cq] and Max [Cq]: Cq threshold values; SD [±Cq]: Cq standard deviation; CV [%Cq]: variance coefficient expressed as percentage of Cq level; SD and CV are indicated in bold.

### Quantitative analysis and determination of expression stability of reference genes

Before stability analysis, the Shapiro–Wilk test was used to determine if the residuals from the ANOVA followed a normal distribution. Data sets that failed the normality test (*P* < 0.05) were transformed using the logarithm transformation. The resulting transformed variables were consistent with a normal distribution (Table 3). To evaluate the reference genes expression stability, five different algorithms were used: BestKeeper, NormFinder, geNorm Excel, geNorm SAS and ΔCt method. For each method and gene a ranking of stability values was obtained and the most stable gene was indicated. The results are summarized on Table 4.

**Table 3:** Normality test (Shapiro-Wilk).

Gene Name	Statistic	df	Sig. (*p*)
ACTA1	0.941	60	0.0130
ACTB	0.588	60	<0.0001
B2M	0.971	60	0.2252*
EEF1	0.984	60	0.7135*
GAPDH	0.7301	60	<0.0001
HMBS	0.933	60	0.0061
HPRT1	0.970	60	0.2047*
LDHA	0.912	60	0.0010
MRPS27	0.981	60	0.5915*
MRPS30	0.866	60	<0.0001
RPL5	0.951	60	0.0317
TFRC	0.955	60	0.0486
UBC	0.933	60	0.0057
COX1	0.819	60	<0.0001

*P-values > 0.05 indicate a normal distribution.

**Table 4:** Results of ranking of thirteen reference genes obtained using five different algorithms and overall rank of candidate reference genes for chicken muscle. Three overall best references genes are indicated in bold.

Gene Name	BestKeeper	GeNorm Excel	GeNorm SAS	NormFinder	ΔCT	Overall rank
**ACTA1**	**0.92 (3)**	**0.67 (3)**	**0.65 (11)**	**0.21 (2)**	**0.82 (2)**	**2**
ACTB	0.88 (5)	0.85 (8)	0.43 (7)	0.39 (9)	0.97 (9)	10
B2M	1.13 (8)	0.95 (9)	0.64 (10)	1.25 (11)	1.25 (11)	12
EEF1	0.91 (4)	0.71 (6)	0.23 (2)	0.27 (5)	0.85 (4)	6
GAPDH	0.91 (4)	0.71 (6)	0.28 (4)	0.23 (3)	0.89 (8)	7
**HMBS**	**0.96 (1)**	**0.66 (2)**	**0.29 (5)**	**0.17 (1)**	**0.80 (1)**	**1**
**HPRT1**	**0.87 (6)**	**0.70 (5)**	**0.22 (1)**	**0.24 (4)**	**0.86 (5)**	**3**
LDHA	0.88 (5)	0.74 (7)	0.26 (3)	0.33 (8)	0.87 (6)	9
MRPS27	0.91 (4)	0.70 (4)	0.34 (6)	0.30 (7)	0.88 (7)	8
MRPS30	0.94 (2)	0.68 (4)	0.46 (8)	0.28 (6)	0.83 (3)	4
RPL5	0.91 (4)	0.68 (4)	0.23 (2)	0.24 (4)	0.82 (2)	5
TFRC	1.88 (9)	1.34 (10)	1.26 (12)	1.87 (12)	1.87 (12)	13
UBC	1.04 (7)	0.54 (1)	0.59 (9)	1.18 (10)	1.18 (10)	11

#### BestKeeper

The best reference genes are those that have the lowest coefficient of variance and standard deviation. Reference genes with SD values >1 are considered not stable and should be avoided [17]. In the present study only UBC had a SD > 1 and thus was removed from the analysis. As a limit of ten genes can be analyzed at once, TFRC and B2M were also not included in the analysis although these genes had SDs below one. In addition, B2M had the highest SD of the remaining genes so was unlikely to be highly ranked as previous describe on the Table 2. The BestKeeper also combines highly correlated genes into an index and the software then compares the correlation between the BestKeeper index and the candidate reference gene to calculate the correlation coefficient (r) value. Genes that have r values close to 1.0 represent the most stable genes. The Pearson correlation coefficient between BestKeeper index and each reference gene gave the highest stability expression to HMBS (r = 0.96) and MRPS30 (r = 0.94); the lowest value was observed to HPRT1 (r = 0.87) followed by ACTA1 (r = 0.88) and LDHA (r = 0.88) (Table 2).

#### NormFinder

**Fig 2:**
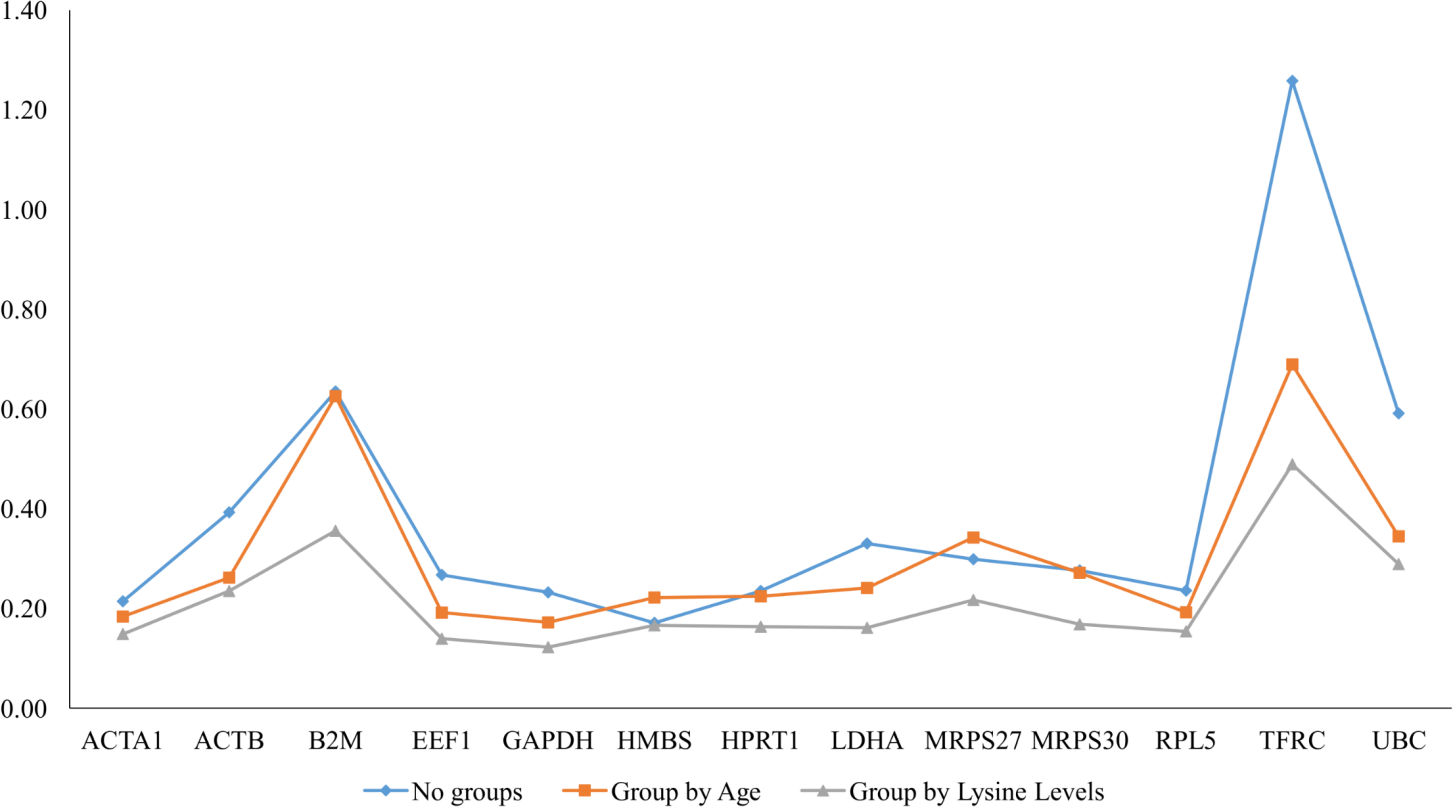
Reference genes stability for all treatments. NormFinder analysis of reference genes in chicken *Pectoralis major* muscle showing stability numbers.

NormFinder is a Microsoft Excel-based Visual Basic application that employs a model-based approach. Genes showing high variance between the analyzed samples should be avoided. A low value, indicating a low combined intra- and inter-group variation, indicates high expression stability [18]. By NormFinder analysis, the most stable reference genes HMBS (= 0.17) followed by ACTA1 (= 0.21) both ranked among the two most stable reference genes; TFRC (= 1.87) and B2M (= 1.25) were defined as the least stable (Table 4 and Fig 2).

#### geNorm

geNorm ranks genes based on their average expression stability (M), and the candidate gene possessing the lowest M value is the most stably expressed gene in that set. To identify reference genes with stable expression, geNorm indicates genes with M values below the threshold of 1.5. We set the threshold of 1.0 to ensure the selection of the most stable genes. In this work, two version of geNorm algorithms were used: geNorm Excel [19] and geNorm SAS [20]. On the geNorm Excel twelve genes exhibited M<1.0 except for TFRC (M = 1.34). Based on M-value, the top three most stable reference genes were UBC (0.54) followed by HMBS (M = 0.66) and ACTA1 (M = 0.67) (Table 4). On the geNorm SAS twelve genes also exhibited M<1.0 except for TFRC (M = 1.26). Based on M-value, the top three most stable reference genes were HPRT1 (M = 0.22), RPL5 and EEF1 (both with values of M = 0.23), and LDHA (0.26) (Table 4).

#### ∆Cq method

The comparative ΔCq method assesses the most stable reference genes by comparing relative expression of “pairs of genes” within each tissue sample reach treatment [21]. If the ΔCq value between pairs of genes remains constant for all samples tested, those reference genes are either stably expressed. The results were very similar to those obtained by BestKeeper and NormFinder, which indicated HMBS (0.80) gene as most stable; but slightly different from the results observed on the geNorm SAS and highly different from geNorm Excel. On the other hand, TFRC was the most variable and had the highest ranking in all algorithms tested (Table 4).

### Determination of the optimal number of reference genes

**Fig 3:**
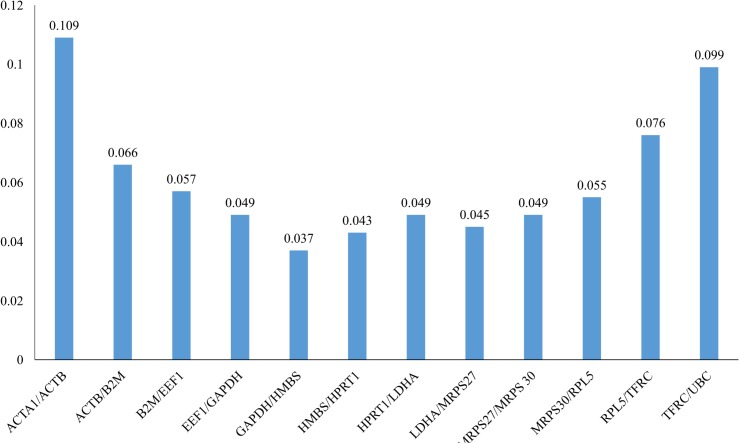
Determination of the Optimal Number of Control Genes for Normalization. This graph show the geNorm Excel pairwise variation (V) analysis. These values were used to determine optimal number of reference genes based on V-value for normalization in RT-qPCR reaction. Each bar represents change in normalization accuracy when stepwise adding more reference gene.

In this work, geNorm Excel was also to predict an optimal number of reference genes. As shown in Fig 3, GAPDH/HMBS pairwise combinations were co-ranked as most stable genes (M = 0.037) and the inclusion of the third reference gene did not contribute significantly to the variation of the normalization factor (V2/3< 0.15). Based on the cut-off value of 0.15, the two most stable reference genes of this dataset would be sufficient for accurate normalization.

### Overall rank of reference genes

**Fig 4:**
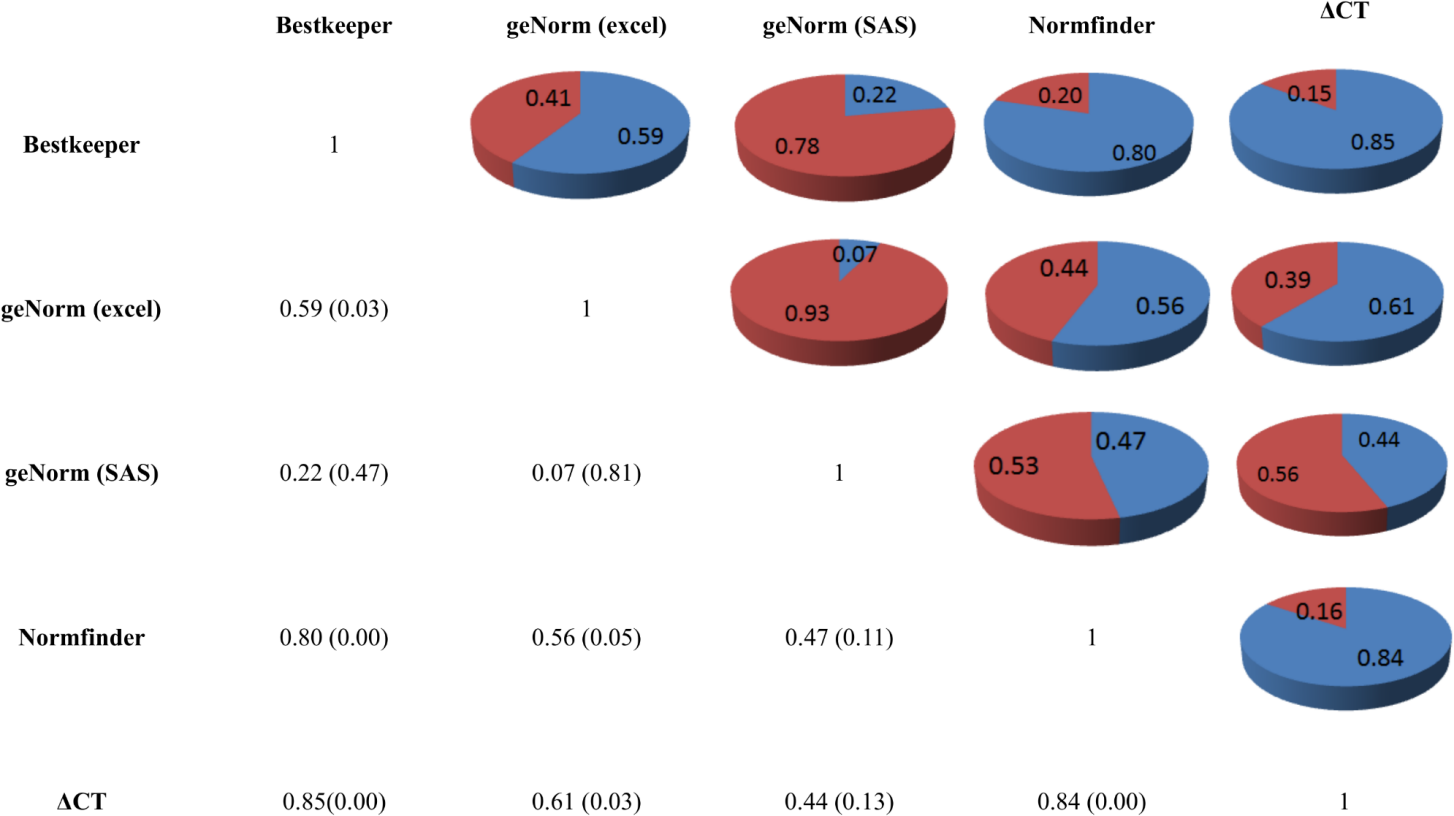
Spearman correlation matrix between five different stability algorithms. A correlation matrix was built between BestKeeper, NormFinder, geNorm Excel, geNorm SAS, and ΔCt method. P-value are described are in parenthesis.

The stability measurements produced by all algorithms were combined to establish a consensus rank of the stable genes. A comparison of the rankings produced by the five approaches revealed differences as a consequence of the type of algorithm applied. The results are summarized in Table 4. The Spearman correlation coefficients was moderate to high except to pairwise combination between geNorm Excel and geNorm SAS (r = 0.07) indicating a high discrepancy between them. The highest correlation was observed between BestKeeper and the ΔCq method (r = 0.85) followed by NormFinder and ΔCq method (r = 0.84), indicating a high concordance in ranking between them (Fig 4). According to overall ranking, the HMBS (1) was ranked as the most stable gene among 13 reference genes (Table 4). Genes ACTA1 (2) and HPRT1 (3) were nearly always the most stably expressed genes when different algorithm was applied for selection of suitable reference gene. As shown in the Table 4, HMBS, ACTA1 and HPRT1 were top ranked by at least two algorithms. Conversely, TFRC (13), B2M (12) and UBC (11) showed the lower stability in expression level by all five algorithms.

### Selection of pairs of reference genes to normalized target gene expression

To determine if there was a co-expression pattern in gene expression between all pairs of the tested genes, Pearson's correlation analysis was performed on gene expression values. The threshold for the correlation among pair of genes was decided as ±0.85. The correlation between all reference genes was moderate to high among the applied algorithms indicating that eight pairwise combinations of genes were co-regulated. The highest correlation was observed between ACTB and EEF1 (r = 0.89) and, the lowest correlation was between the TFRC and MRPS30 (r = 0.00) (Table 5).

According to results describe above (the overall rank, the Pearson coefficient of correlation and the optimal number of reference genes) HMBS and HPRT1 was the most stable of pair of reference genes and choose to normalize the target gene expression.

**Table 5:** Pearson correlation matrix visualizing reference genes ranked by five different stability tests (BestKeeper, NormFinder, geNorm Excel, geNorm SAS, and ΔCt method. p-value are described are in parenthesis. Significative values are indicated in bold.

	GAPDH	ACTB	MRPS27	MRPS30	EEF1	HMBS	TRFC	RPL5	LDHA	UBC	B2M	ACTA1	HPRT1
**GAPDH**	1												
**ACTB**	-0.04242 (0.7653)	1											
**MRPS27**	-0.07199 (0.6120)	0.76757 (<.0001)	1										
**MRPS30**	-0.11391 (0.4214)	0.67690 (<.0001)	0.75617 (<.0001)	1									
**EEF1**	-0.01464 (0.9180)	**0.89588 (<.0001)**	**0.85383 (<.0001)**	0.69175 (<.0001)	1								
**HMBS**	0.05856 (0.6801)	**0.87375 (<.0001)**	0.81317 (<.0001)	0.68205 (<.0001)	**0.86336 (<.0001)**	1							
**TRFC**	0.05672 (0.6896)	0.36186 (0.0084)	-0.01783 (0.9001)	**-0.00355 (0.9801)**	0.19983 9 (0.15550)	0.24023 9 (0.0863)	1						
**RPL5**	0.07147 (0.6146)	0.73665 (<.0001)	0.82305 (<.0001)	0.63609 9 (<.0001)	**0.86880 (<.0001)**	**0.87448 (<.0001)**	0.13456 (0.3416)	1					
**LDHA**	0.04835 (0.7336)	0.64899 (<.0001)	0.79144 (<.0001)	0.57684 9 (<.0001)	0.68718 (<.0001)	0.84655 (<.0001)	-0.01101 (0.9383)	0.77235 9 (<.0001)	1				
**UBC**	0.15702 (0.2663)	0.38387 (0.0050)	0.47499 (0.0004)	0.26974 (0.0531)	0.44685 (0.0009)	0.51456 (<.0001)	0.08761 (0.5368)	0.44447 (0.0010)	0.52439 9 (<.0001)	1			
**B2M**	0.11180 (0.4301)	0.68587 (<.0001)	0.32674 (0.0181)	0.35673 (0.0094)	0.55599 (<.0001)	0.73018 (<.0001)	0.58638 (<.0001)	0.55885 (<.0001)	0.43955 (0.0011)	0.29072 (0.0365)	1		
**ACTA1**	0.03919 (0.7827)	0.68697 (<.0001)	0.82076 (<.0001)	0.59736 (<.0001)	0.74109 (<.0001)	**0.85441 (<.0001)**	0.18722 (0.1838)	**0.87033 (<.0001)**	**0.88338 (<.0001)**	0.57594 (<.0001)	0.54729 (<.0001)	1	
**HPRT1**	0.04859 (0.7323)	0.72966 (<.0001)	0.76918 (<.0001)	0.64478 (<.0001)	0.78810 (<.0001)	**0.80925 (<.0001)**	0.26315 (0.0595)	0.76054 (<.0001)	0.70318 (<.0001)	0.65141 (<.0001)	0.55628 (<.0001)	0.80996 (<.0001)	1

### Evaluation and validation of selected reference genes using MT-CO2

**Fig 5:**
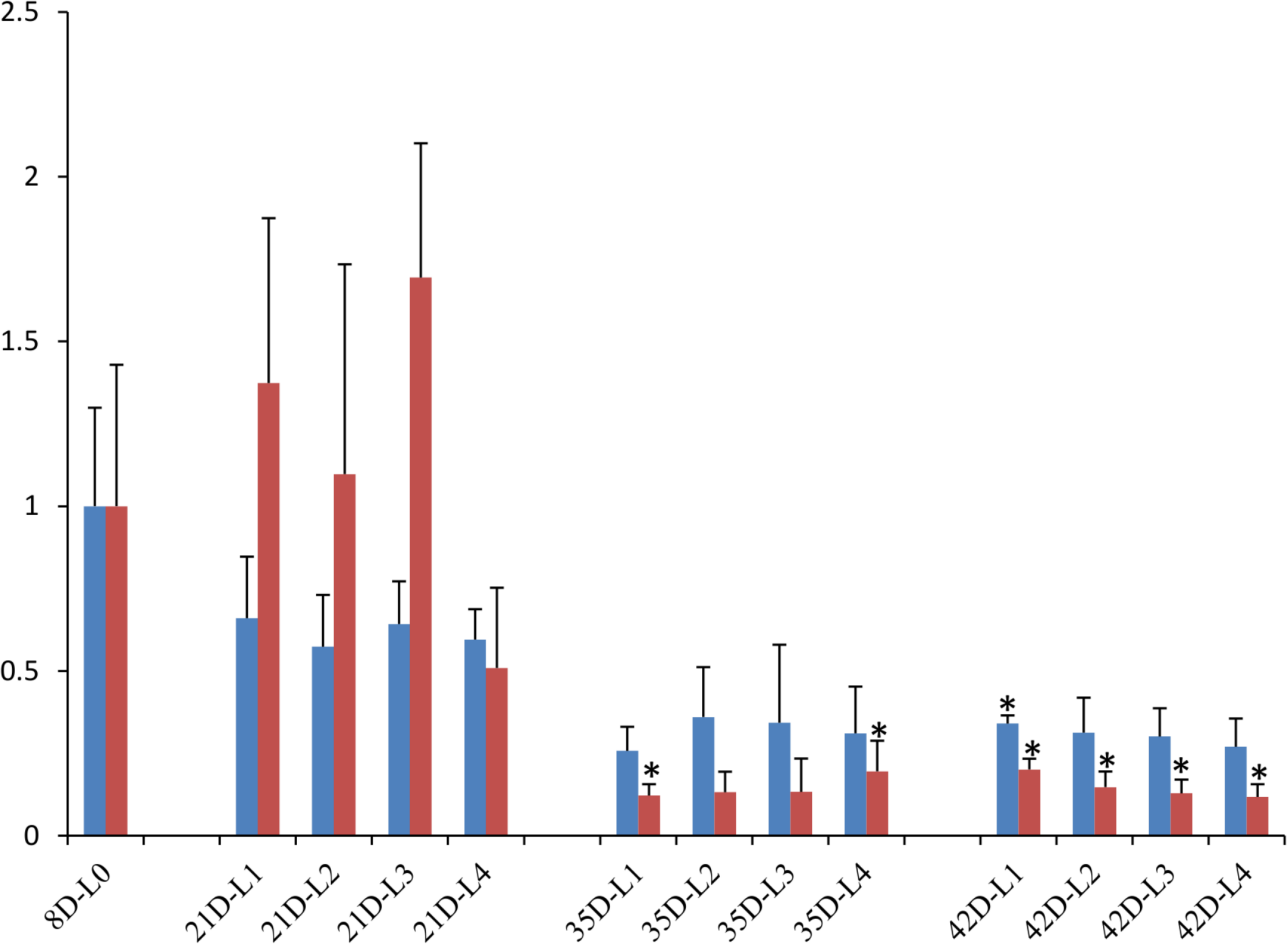
Relative expression of this MT-CO2 target gene when normalized with poor ranked reference genes and the best reference genes using Pfaffl method. This Figure shows the effect of incorrect selection of reference gene in the gene expression of target gene. Two best ranked reference genes (HMBS and HPRT1) and the two poor ranked reference genes (TFRC and B2M). The results also include standard errors (SE_R) and two-tailed P values by Student's t test. A single asterisk indicates P-value < 0.05.

Two different sets of reference genes were used to normalize gene expression. The best-ranked genes HMBS and HPRT1 formed the first set, and a second set was formed by TFRC and B2M that showed the worst values (Table 4). By using the geometric mean of gene expression of best reference genes (HMBS and HPRT1) were observed a significant difference in expression for MT-CO2 in skeletal muscle of chicken slaughtered at 42 d post hatch compared to control group (Ratio = 0.340, *P* = 0.025; Fig 5). However, when MT-CO2 was normalized using lower ranked reference genes (TFRC and B2M) a significant decrease of gene expression can be detected (Ratio = 0.201, *P* = 0.032; Fig 5), indicating that TFRC and B2M are inappropriate to be used as reference genes. In addition, results showed when this worst reference gene set is used MT-CO2 expression was also significantly decreased in skeletal muscle of chicken slaughtered at 35 d post hatch (Fig 5).

## Discussion

The use of different reference genes to evaluate relative expression data has an important impact on the final normalized results. Here, to improve the gene expression analysis by RT-qPCR in skeletal muscle of domestic chicken, we evaluated the suitability of 13 common reference genes in *Pectoralis major* muscle obtained from chickens in different development periods post hatch and under different nutritional diets Then, their expression was analyzed by using five different algorithms: BestKeeper, NormFinder, GeNorm Excel, GeNorm SAS and ∆Ct method.

Our study revealed considerable variation in the correlation among the applied algorithms. We expected that geNorm Excel e geNorm SAS would show the most consistency compared with other algorithms in terms of ranking of reference genes. For example, the ACTA1 gene was within the second or third the most stable genes in four of the five algorithms used in this study, except to geNorm SAS. Although they are the same algorithm, geNorm Excel use the real efficiency calculated by standard curve and accept PCR efficiency calculated above 100%. On the other hand, geNorm SAS use the primer efficiency with maximum value up to 100% (Table 6), which explains the divergent results among both algorithms.

**Table 6:** Comparative primer efficiency as calculated by geNorm Excel and geNorm SAS.

	geNorm Excel		geNorm SAS	
Gene symbol	Primer efficiency	R2	Primer efficiency	R2
ACTA1	1.03	0.999	0.95	0.999
ACTB	1.01	0.997	0.99	0.995
B2M	0.91	0.996	0.91	0.994
EEF1	1.00	0.992	1.00	0.990
GAPDH	1.00	0.994	0.91	0.994
HMBS	1.00	0.998	1.00	0.997
HPRT1	0.97	0.999	0.98	0.999
LDHA	1.03	0.993	0.97	0.991
MRPS27	0.96	0.996	0.96	0.993
MRPS30	1.02	0.999	0.92	0.996
RPL5	1.04	0.996	0.96	0.995
TFRC	0.98	0.991	0.98	0.989
UBC	1.05	0.970	0.95	0.962
MT-CO2	1.00	0.997	1.00	0.999

Ranking inconsistence was also observed between others algorithms. An interesting fact is that UBC (1) is clearly pointed as more stable genes in geNorm Excel results but only ranked the ninth in geNorm SAS and seven in overall rank. Moreover, despite the relatively high correlation between the BestKeeper and the ΔCt algorithms (r = 0.85), the application of these two algorithms delivered identical ranking in only 2 out of the investigated 13 reference genes while between NormFinder and ΔCt algorithms (r = 0.84) seven genes were ranked in the same position. However, Beekeeper ranked four genes (EEF1, GAPDH, MRPS27 and RPL5) in the same position (4) which may explain this apparent inconsistency between this algorithm and others ones. Therefore, based on our observations we recommend the use of at least Δct, NormFinder and geNorm Excel algorithms for selecting the most stably expressed reference genes.

Although this work was not designed to measure the effect of lysine supplementation or periods post hatch in the reference gene expression, it appears that treatments affects the stability rankings of some specific reference genes. Our data showed that expression stability varied considerably among the 13 reference genes tested in chicken *Pectoralis major* muscle. This was particularly the case with TFRC and B2M, and this would therefore be completely inappropriate to use as reference genes. From all genes evaluated, those that scored highest in their requirements as reference genes were HMBS, ACTA1 and HPRT1. These three genes fulfilled most criteria as suitable reference genes in that it was strongly stable and displayed minimal fluctuation. For an accurate measure of expression levels normalization by multiple reference genes is suggested. However, the strongest correlation was observed between HMBS and ACTA1 expression. Therefore, we selected HMBS and HPRT1 or ACTA1 and HPRT1 as the most appropriated pair of reference genes. HMBS gene has already shown stability in, salmon muscle [22] and bovine muscle [23]. Interestingly, ACTB was ranked as the tenth reference gene and clearly performed with lower stability compared to other reference genes. This result is in accordance with previous report that observed variations in ACTB expression throughout RT-qPCR experiments [8].

Our results revealed an appropriate reference gene set to normalize the MT-CO2 target gene. The *Mitochondrially encoded cytochrome c oxidase II* is a protein-coding gene component key of the respiratory chain that catalyzes the reduction of oxygen to water and ATP production. Our results clearly demonstrated that using a commonly uncorrected reference gene without verification would yield to misleading gene expression. The results of relative gene expression analysis demonstrate the importance of normalizing to appropriate reference gene set that is unaffected by experimental condition may differ in the expression stability of the reference genes.

A large number of RT-qPCR studies have been carried out concerning the validation of reference genes in birds [11]. However, it has been difficult to find information on appropriate reference genes for use in chicken. To our knowledge, this is the first study that investigated different candidate reference genes for gene expression analyses and seems to be useful in guiding researchers performing gene expression analyses in this specie.

## Conclusion

We present experimentally validated comparisons of reference genes for the normalization of RT-qPCR expression data in studies involving lysine supplementation in chicken at different days post hatch. Our findings highlighted HMBS and HPRT1 as the most stably expressed genes that can be used for normalization of expression data in *Pectoralis major* muscle of chickens. The identification of these two reference genes seems to be useful in guiding researchers performing gene expression analyses in chicken *Pectoralis major* muscle and will benefit future expression studies by providing stability in gene expression analyses.

## Supporting Information

S1 File Raw data of Ct values according to experimental treatments. (XLS)
